# DecentTree: scalable Neighbour-Joining for the genomic era

**DOI:** 10.1093/bioinformatics/btad536

**Published:** 2023-08-31

**Authors:** Weiwen Wang, James Barbetti, Thomas Wong, Bryan Thornlow, Russ Corbett-Detig, Yatish Turakhia, Robert Lanfear, Bui Quang Minh

**Affiliations:** China National GeneBank, BGI Research, Shenzhen 518083, China; School of Computing, College of Engineering and Computer Science, Australian National University, Canberra, ACT 2601, Australia; Ecology and Evolution, Research School of Biology, College of Science, Australian National University, Canberra, ACT 2601, Australia; Genomics Institute, University of California Santa Cruz, Santa Cruz, CA 95064, United States; Biomolecular Engineering, University of California Santa Cruz, Santa Cruz, CA 95064, United States; Genomics Institute, University of California Santa Cruz, Santa Cruz, CA 95064, United States; Biomolecular Engineering, University of California Santa Cruz, Santa Cruz, CA 95064, United States; Electrical and Computer Engineering, University of California San Diego, La Jolla, CA 92093, United States; Ecology and Evolution, Research School of Biology, College of Science, Australian National University, Canberra, ACT 2601, Australia; School of Computing, College of Engineering and Computer Science, Australian National University, Canberra, ACT 2601, Australia

## Abstract

**Motivation:**

Neighbour-Joining is one of the most widely used distance-based phylogenetic inference methods. However, current implementations do not scale well for datasets with more than 10 000 sequences. Given the increasing pace of generating new sequence data, particularly in outbreaks of emerging diseases, and the already enormous existing databases of sequence data for which Neighbour-Joining is a useful approach, new implementations of existing methods are warranted.

**Results:**

Here, we present DecentTree, which provides highly optimized and parallel implementations of Neighbour-Joining and several of its variants. DecentTree is designed as a stand-alone application and a header-only library easily integrated with other phylogenetic software (e.g. it is integral in the popular IQ-TREE software). We show that DecentTree shows similar or improved performance over existing software (BIONJ, Quicktree, FastME, and RapidNJ), especially for handling very large alignments. For example, DecentTree is up to 6-fold faster than the fastest existing Neighbour-Joining software (e.g. RapidNJ) when generating a tree of 64 000 SARS-CoV-2 genomes.

**Availability and implementation:**

DecentTree is open source and freely available at https://github.com/iqtree/decenttree. All code and data used in this analysis are available on Github (https://github.com/asdcid/Comparison-of-neighbour-joining-software).

## 1 Introduction

Neighbour-Joining (NJ) (Saitou and Nei 1987) is perhaps the most widely used distance-based algorithm for inferring phylogenies. Its success results from its simplicity and computational efficiency: NJ takes a fraction of the time required by other popular approaches (e.g. Maximum Likelihood and Bayesian methods) and is known to perform well in terms of speed and accuracy for large alignments with low sequence divergence (Yang and Rannala 2012). Although other approaches such as Maximum Likelihood and Bayesian approaches perform better than NJ in many situations, the properties of NJ make it an attractive method for a range of applications, including generating starting trees for more computationally expensive approaches, generating rapid trees from large alignments such as SARS-CoV-2 (McBroome *et al.* 2021), ribosomal RNA (Quast *et al.* 2013), and DNA barcodes (Ratnasingham and Hebert 2007), and NJ is also widely used to provide guide trees for alignment algorithms (Thompson *et al.* 1994, Edgar 2004). Despite its utility, even the fastest existing implementation of NJ, RapidNJ (Simonsen *et al.* 2008), does not scale well for alignments containing more than 10 000 sequences, and there are no scalable implementations of NJ algorithms that are written as libraries that can be easily incorporated into other software. DecentTree seeks to address these limitations.

## 2 Implementation

DecentTree is an optimized and parallel C++ implementation of NJ and BIONJ (Gascuel 1997). DecentTree uses the Vector Class Library (VCL; https://github.com/vectorclass) and the multithreading OpenMP to parallelize the computations. Moreover, we reimplemented the RapidNJ algorithm to optimize memory access patterns and reduce CPU cache miss. The VCL version of NJ and BIONJ implementation in DecentTree is denoted with ‘-V’ suffix (NJ-V and BIONJ-V), whereas the RapidNJ reimplementation is denoted with ‘-R’ suffix (NJ-R and BIONJ-R). Like other standard implementations of NJ/BIONJ, DecentTree has a worst-case time complexity of O(*n*^3^), where *n* is the number of taxa. But we expect DecentTree to be faster in most cases due to the highly optimized code.

DecentTree is designed as both a stand-alone program and a header-only library that can be easily integrated into other phylogenetic software packages. DecentTree uses C++ template classes that allow for flexible configuration of the runtimes such as choosing between single or double precision arithmetic. As input, DecentTree accepts either a distance matrix in Phylip format or a multiple sequence alignment in common formats such as Phylip or Fasta. When users provide a sequence alignment, DecentTree computes the Jukes-Cantor pairwise distance matrix (Jukes and Cantor 1969) from the alignment. As output, DecentTree reconstructs a distance-based tree in Newick format. We checked the correctness of our implementations on simulated data with 100 taxa and alignment lengths ranging from 1000 to 100 000 sites (Supplementary Fig. S1) and assessed the code quality of DecentTree using SoftWipe (Zapletal *et al.* 2021). The overall score of DecentTree was 5.7, slightly higher than the average score of 5.6 for software evaluated by Zapletal *et al.* (2021).

## 3 Benchmarking

We compared the performance of the four implementations of the NJ and BIONJ algorithms in DecentTree (NJ-R, NJ-V, BIONJ-R, BIONJ-V) to implementations of the same algorithms in four other software implementations which take distance matrices as input [the BIONJ algorithm implemented in the original BIONJ software, the NJ and BIONJ algorithms implemented in FastME v2.1.6.2 (Lefort *et al.* 2015), and the NJ algorithms implemented in Quicktree v2.5 (Howe *et al.* 2002) and RapidNJ v2.3.2] and one other implementation which takes a sequence alignment as input [FastTree (Price *et al.* 2009, Price *et al.* 2010)]. To do this, we analysed the simulated data we used to check DecentTree (see above), and three challenging empirical datasets: a SARS-CoV-2 alignment (COVID19 datasets) (https://github.com/bpt26/parsimony, accessed on 11 May 2021) (McBroome *et al.* 2021) and two high-quality ribosomal RNA v138.1 datasets from the SILVA database (Quast *et al.* 2013), the small subunit (SSU_NR99) and the large subunit (LSU_NR99) datasets. To examine performance across a range of dataset sizes, we randomly subsampled seven subsets of 1000, 2000, 4000, 8000, 16 000, 32 000, and 64 000 sequences from each dataset using Seqtk version 1.3-r116-dirty (https://github.com/lh3/seqtk). For the comparisons which use a distance matrix as input we first computed the distance matrix for each subset using DecentTree and then ran all software using these distance matrices as input (command see Supplementary Information). For the programmes that support multiple-threading (DecentTree, FastME, RapidNJ, and FastTree), we benchmarked them with 1 thread and 32 threads. We set the maximum wall-clock time to 12 h and the maximum memory limit to 500 Gb.

These settings resulted in 378 analyses for using distance matrices as input: 21 data subsets × 9 implementations (4 for DecentTree, 2 for FastME, and 1 for BIONJ/Quicktree/RapidNJ) × 2 thread counts; and 210 analyses using multiple alignments as input: for 21 data subsets × 5 implementations (4 for DecentTree and 1 for FastTree) × 2 thread counts. We recorded the wall-clock time and peak memory usage of each analysis on a server with 256 CPUs of 2.5 GHz and 1 Tb RAM. To compare the resulting trees, we computed their log-likelihoods using IQ-TREE (Nguyen *et al*., 2015) under the GTR+G model.

## 4 Results

Analyses of simulated data show that all algorithms in DecentTree, BIONJ, FastME, and RapidNJ performed well, but Quicktree and FastTree (using the NJ anlaysis only) performed poorly in terms of the Robinson–Foulds (RF) distance of the estimated trees to the true trees (Supplementary Fig. S9).

**Figure 1.**
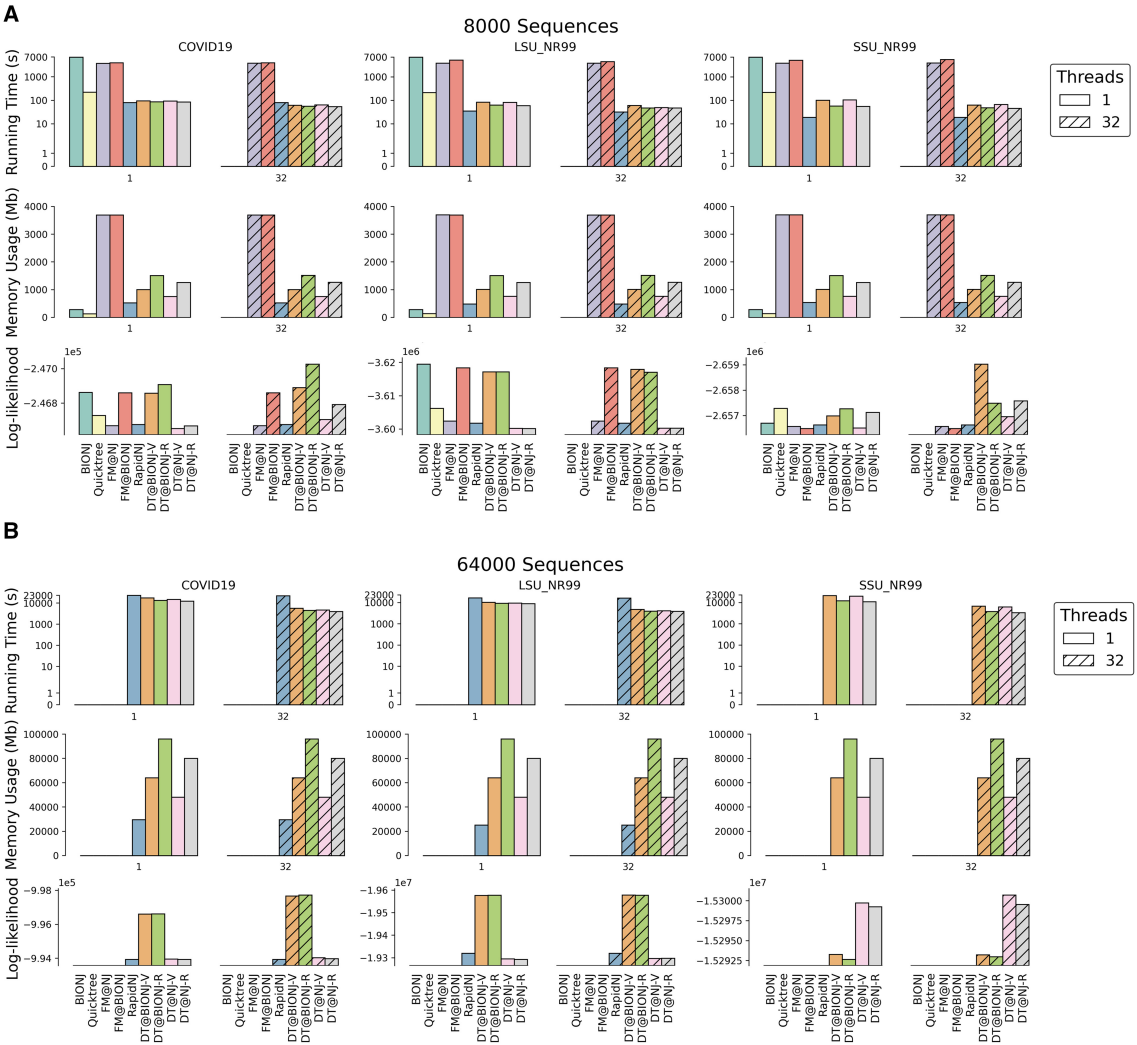
The comparison of different implementations on the 8000 (A) and 64 000 (B) sequence subsets of COVID19, LSU_NR99, and SSU_NR99 datasets. BIONJ and Quicktree do not support multithreading.

Analyses of empirical data show that for analyses which started with distance matrices, DecentTree was the only implementation that completed every analysis within 12 h (Supplementary Fig. S2) and was the fastest implementation on the larger subsets of each of the three datasets. On the smaller subsets of each dataset (≤8000 sequences), RapidNJ tended to be the fastest implementation, although the absolute differences in execution time versus the fastest DecentTree algorithm were small (0.5–36 s when RapidNJ was faster, while for the COVID19 dataset with 8000 sequences and 32 threads available DecentTree was 26 s faster) (Fig. 1 and Supplementary Figs S3–S5). DecentTree tended to be the fastest implementation on the larger subsets of each dataset, particularly when using multiple threads, although this does come at the cost of using ∼1.5× to ∼3× more memory than RapidNJ (Fig. 1 and Supplementary Figs S6 and S7). On the largest subsets we analysed (64 000 sequences), DecentTree was 2.9 and 5.1 h (1.8 and 5.6 fold) faster than RapidNJ for the COVID19 dataset with 1 and 32 threads, respectively (Fig. 1 and Supplementary Table S1). For the LSU_NR99 dataset, DecentTree was 2.2 and 3.5 h (1.9 and 4.2 fold) faster than RapidNJ with 1 and 32 threads, respectively; and DecentTree was the only software able to complete the analysis of the 64 000 sequence SSU_NR99 dataset in under 12 h (RapidNJ quit without producing any output for this dataset, and we were unable to determine why). The performance of the four DecentTree algorithms (NJ-R, NJ-V, BIONJ-R, BIONJ-V) differed modestly, but NJ-R tended to be the fastest, particularly on the larger subsets, while NJ-V tended to be the most memory efficient. Likelihood analysis shows that the different implementations produce trees that can differ somewhat in their fit to the data. For example, the different implementations produce trees that differ by 30–2681 units of log-likelihood on COVID19 datasets. NJ algorithms tended to produce trees with higher likelihoods than BIONJ algorithms for COVID19 and LSU_NR99, but worse trees for SSU_NR99 (Fig. 1 and Supplementary Figs S3–S7). RF distances (Supplementary Figs S8–S14) show that the tree topologies inferred by DecentTree and other implementations are sometimes different from each other, e.g. by 3.43%–23.51% on 8000 sequences datasets. These large differences are driven by the very large proportion of very short branches in these trees (e.g. more than 70% of branches in the 64 000 sequence COVID tree represent <1 substitution. Supplementary Fig. S15). Because the alignments lack information to resolve these branches, most are resolved effectively at random, leading to large normalized RF distances.

We compared DecentTree to FastTree when inferring a tree from an alignment (the results above pertain to trees inferred from distance matrices). The results (Supplementary Fig. S16) showed that when using one thread, DecentTree is generally slower than FastTree, e.g. by 1.4–1.9 times on 64 000 sequences, but this is reversed with 32 threads (e.g. DecentTree is 2.7–14.1 times faster than FastTree on 64 000 sequences). DecentTree always consumed more RAM than FastTree. For instance, DecentTree NJ-V and FastTree required ∼50 GB and ∼17 GB RAM on 64 000 sequences, respectively. This memory footprint means that DecentTree may not be applicable for datasets with millions of sequences. Likelihood analysis shows that Decenttree NJ-V produces trees with a higher likelihood on COVID19 datasets.

To test the influence of the types of topological move used by different software on the inferred trees, we built additional trees on the 4000 sequence subsets (COVID19, LSU_NR99, and SSU_NR99) using FastME (SPRs), FastTree (NNIs), and DecentTree (default setting), and compared the log-likelihoods of the resulting trees using a GTR+G model in IQ-TREE (Supplementary Fig. S17). The result shows that FastME (SPRs) has the best log-likelihood on COVID19 and SSU_NR99 subsets, whereas FastTree (NNIs) has the best log-likelihood on the LSU_R99 subset. Log-likelihood differences between the trees inferred by DecentTree are at most 380 to 51 240 units, while these numbers are from 0 to 65 738 for FastTree.

## 5 Conclusions

DecentTree allows users to quickly estimate very large NJ trees with a range of algorithms. In addition, because DecentTree is implemented as both stand-alone software and a header-only library, it is easy to incorporate it into other software. This will help to ensure that future improvements in NJ algorithms can be seamlessly integrated into other software and pipelines.

## Supplementary Material

btad536_Supplementary_Data

## Data Availability

DecentTree is open source and freely available at https://github.com/iqtree/decenttree. All code and data used in this analysis are available on Github (https://github.com/asdcid/Comparison-of-neighbour-joining-software).
