# Supplementary material for: DecentTree: scalable Neighbour-Joining for the genomic era
Source: Bioinformatics. 2023 Aug 31;39(9):btad536. doi: 10.1093/bioinformatics/btad536 (PMC10491953; doi:10.1093/bioinformatics/btad536)
Supplement: btad536_Supplementary_Data [file btad536_supplementary_data.docx]

**Supplementary information**

Table of Contents

[1. Supplementary code 1](#_Toc140958482)

[2 Supplementary Figures 2](#_Toc140958483)

[3 Supplementary Table 12](#_Toc140958484)

# 1. Supplementary code

1.1 Distance matrix

# inputFile is the multiple sequence alignment file

decentTree -fasta $input File -no-matrix -dist-out $outputFile -t NONE -no-out truncate-name-at " " -no-banner -nt $threads

1.2 Decenttree

# method is BIONJ-R or BIONJ-V or NJ-R or NJ-V

# inputFile is the distance matrix

decentTree -in $inputFile -nt $threads -t $method -out $outputFile

1.3 FastMe

# method is BIONJ or NJ

# inputFile is the distance matrix

fastme -m $method -T $threads -i $inputFile -o $outputFile

1.4 RapidNJ

# inputFile is the distance matrix

rapidnj $inputFile -i pd -c $threads -x $outputFile

1.5 Quicktree

# inputFile is the distance matrix

quicktree -in m -out t $inputFile 1> $outputFile

1.6 BIONJ

# inputFile is the distance matrix

BIONJ $inputFile $outputFile

1.7 FastTree

# inputFile is the multiple sequence alignment file

export OMP_NUM_THREADS=$threads

FastTreeMP -nt -nome -fastest -nosupport -noml -out $outputFile $inputFile

1.8 Compare the log likelihood of each output tree

# alingmentFile is the multiple sequence alignment file

# treeFile is the tree output of each combination

iqtree -s $alignmentFile -te $treeFile -m GTR+G --prefix $outputPrefix

1.9 Compare the Robinson-Foulds (RF) distance between the different trees inferred on each dataset

# treeFile is the tree output of each combination

iqtree2 -rf $treeFile1 $treeFile2

1.10 Compare the performance of different implementations with NNIs/SPRs

# using the 4,000 sequence alignment as the test sample, and compare three implementation: DecentTree, FastME and FastTree

# run FastME with SPRs

fastme --spr -m $method -T $threads -i $inputFile -o $outputFile

# run FastTree with NNIs

fastTreeMP -nt -nni -noml -out $outputFile $inputFile

# run DecentTree with default (no NNIs or SPRs)

decentTree -in $inputFile -nt $threads -t $method -out $outputFile

# 2 Supplementary Figures


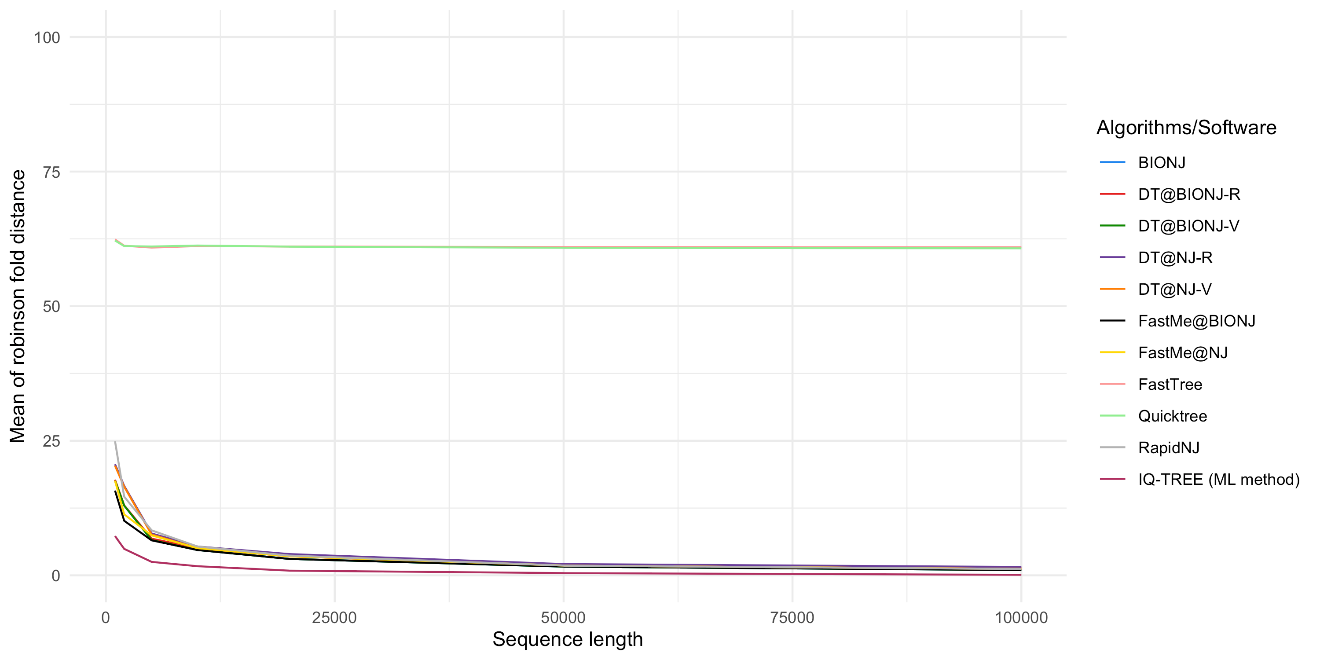


Supplementary Figure S1. Testing the accuracy of different algorithms and software tools by using simulated data sets. The charts show the average of the Robinson fold distances between the estimated trees and the true trees on the data sets simulated under the JC model and trees with 100 tips. A total of 700 data sets were simulated, 100 data sets for each of the following sequence lengths: 1k, 2k, 5k, 10k, 20k, 50k, and 100k. All algorithms and software, except Quicktree and FastTree, performed well. Note that the performance of IQ-TREE is shown for reference only. Its performance is the best due to the maximum likelihood method.


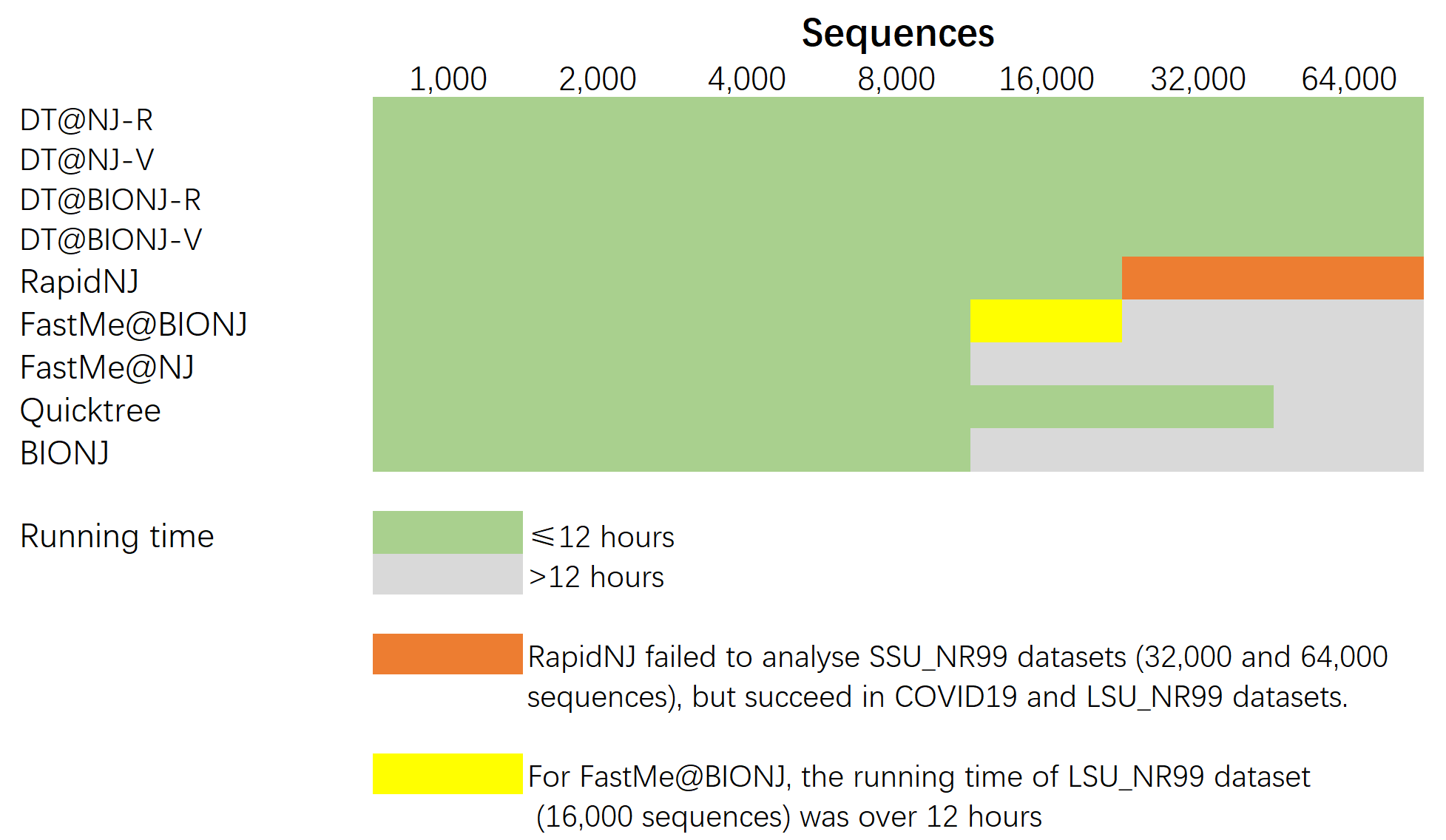


Supplementary Figure S2. The running time (wall-clock) of each implementation. Green denotes that analyses on all three datasets (COVID19, LSU_NR99, and SSU_NR99) ran in under 12 hours, grey denotes that running times were all over 12 hours, and orange and yellow denote mixed results explained in the legend.


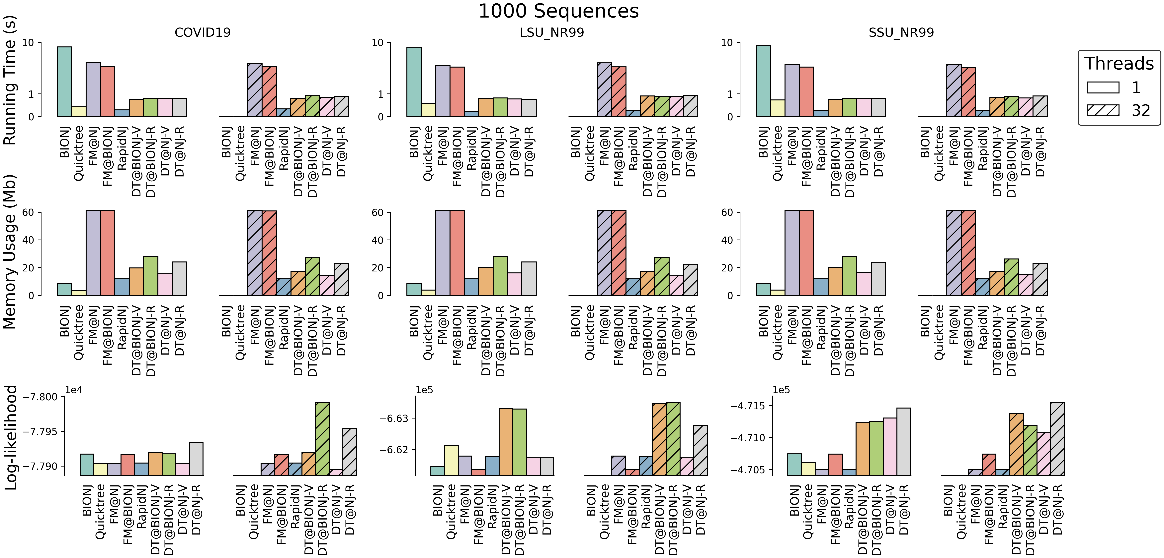


Supplementary Figure S3. The comparison of different implementations in the 1,000 sequence subset of COVID19, LSU_NR99, and SSU_NR99 datasets. BIONJ and Quicktree do not support multi-threading.


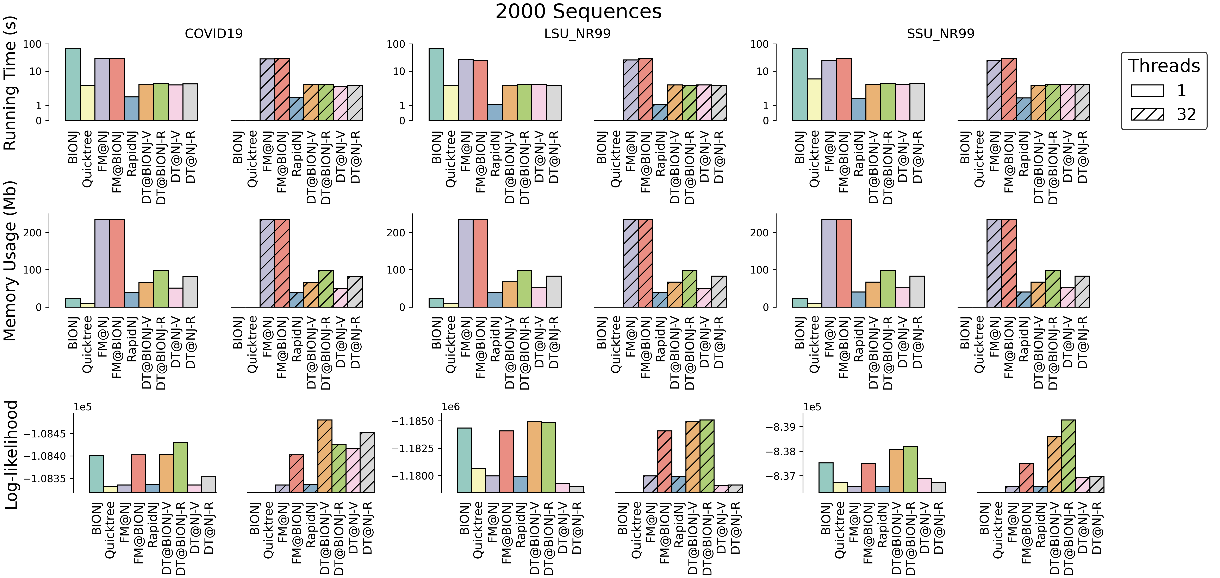


Supplementary Figure S4. The comparison of different implementations in the 2,000 sequence subset of COVID19, LSU_NR99, and SSU_NR99 datasets. BIONJ and Quicktree do not support multi-threading.


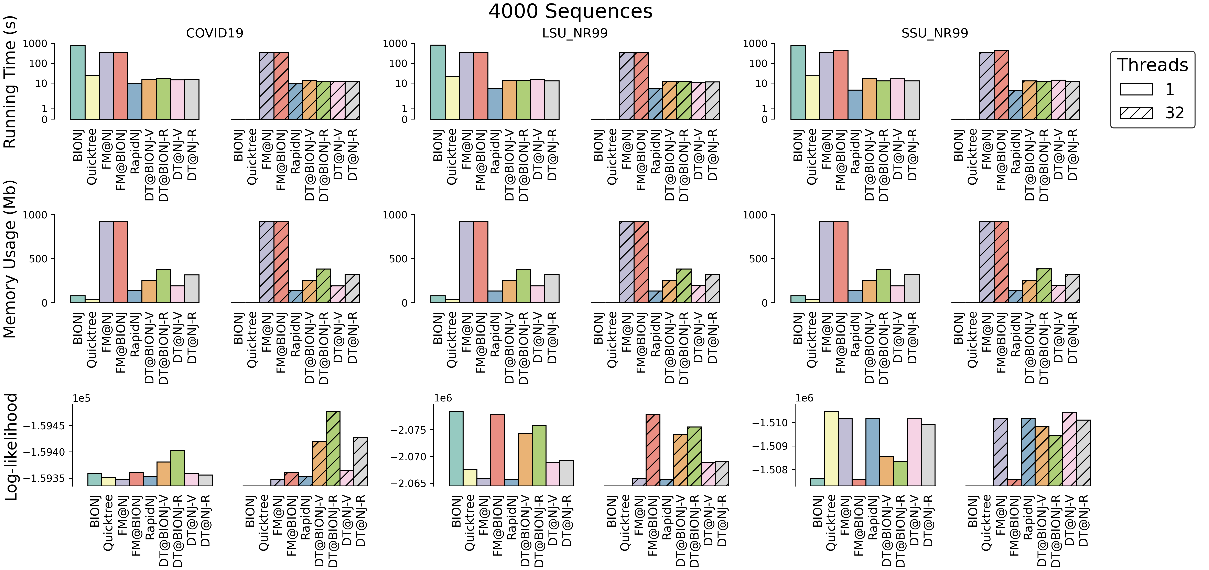


Supplementary Figure S5. The comparison of different implementations in the 4,000 sequence subset of COVID19, LSU_NR99, and SSU_NR99 datasets. BIONJ and Quicktree do not support multi-threading.


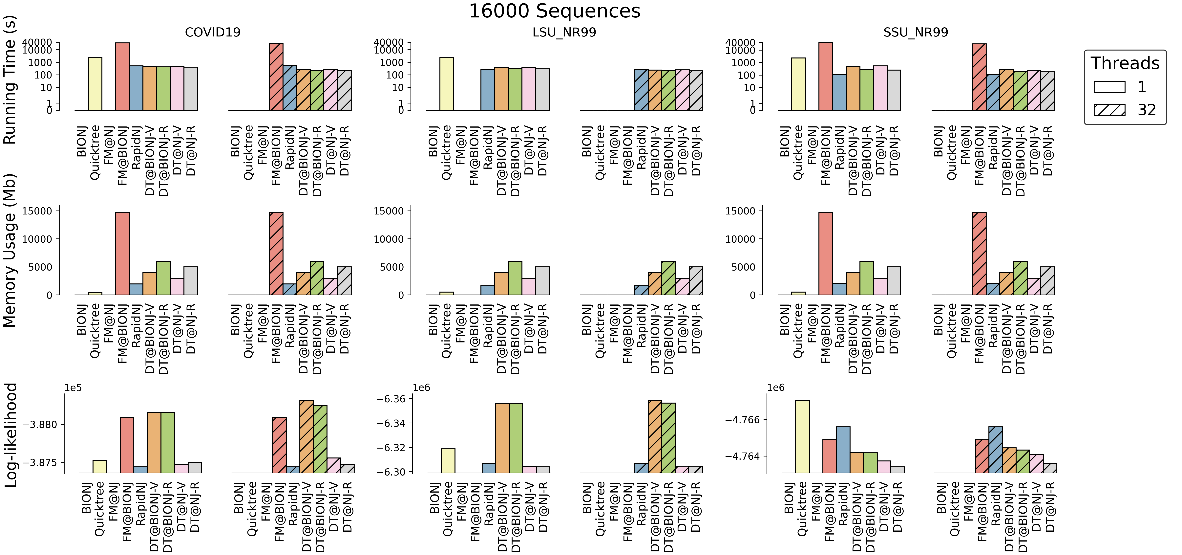


Supplementary Figure S6. The comparison of different implementations in the 16,000 sequence subset of COVID19, LSU_NR99, and SSU_NR99 datasets. BIONJ and Quicktree do not support multi-threading. The empty results of other implementations are because their running times were over 12 hours.


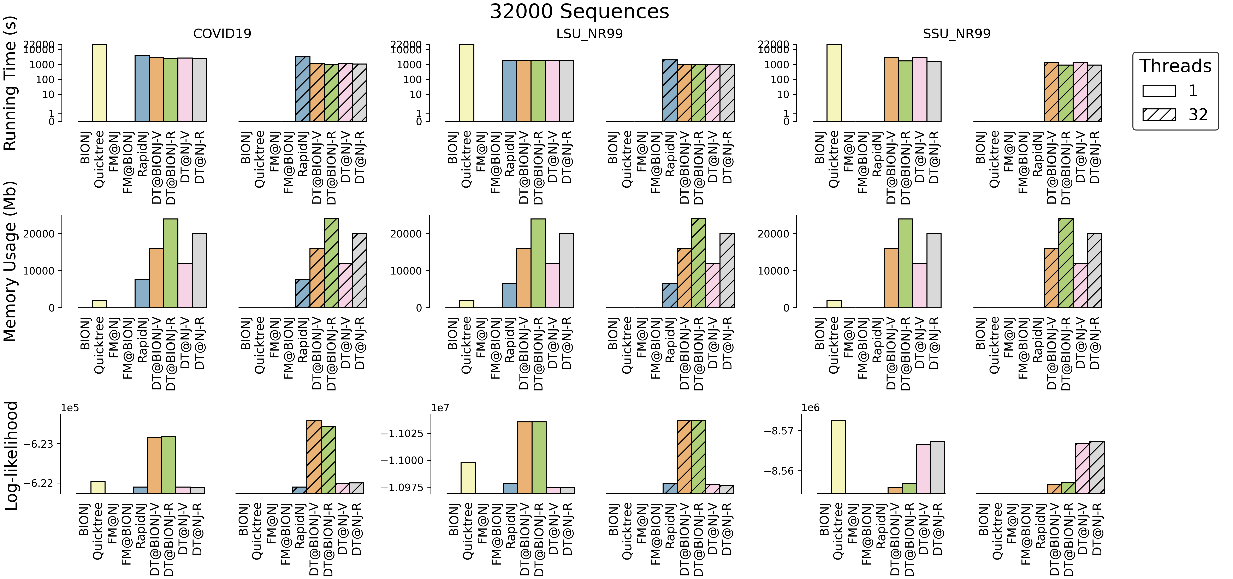


Supplementary Figure S7. The comparison of different implementations in the 32,000 sequence subset of COVID19, LSU_NR99, and SSU_NR99 datasets. BIONJ and Quicktree do not support multi-threading. RapidNJ failed to analyse the SSU_NR99 32,000 sequence subset for unknown reasons. The empty results of other implementations are because their running times were over 12 hours.


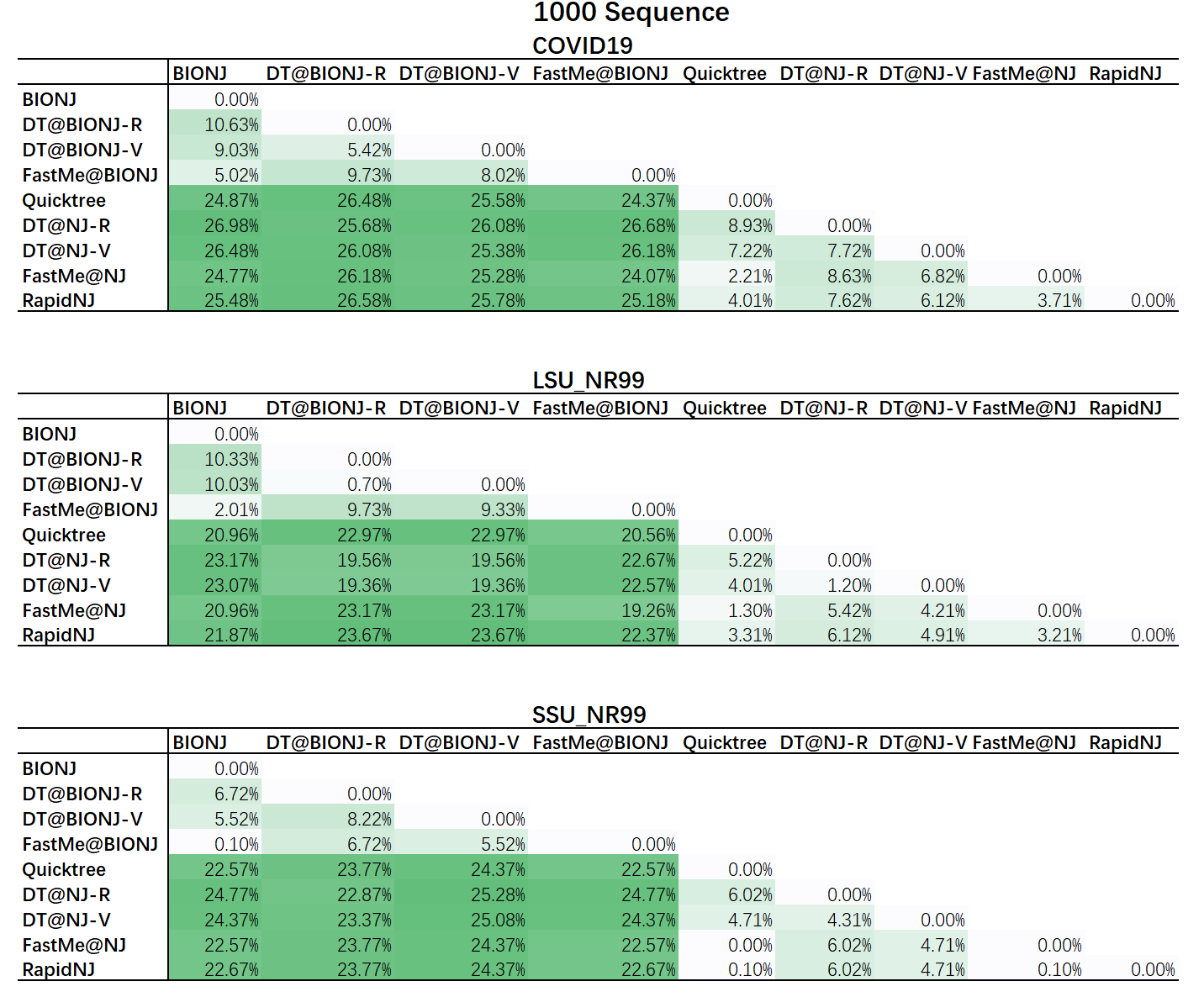


Supplementary Figure S8. The Robinson-Foulds (RF) distances between different trees on the 1,000 sequence datasets. The RF distances were normalized by dividing the current RF by 2(n-3), where n = #taxa. The implementations with higher differences are colored green.


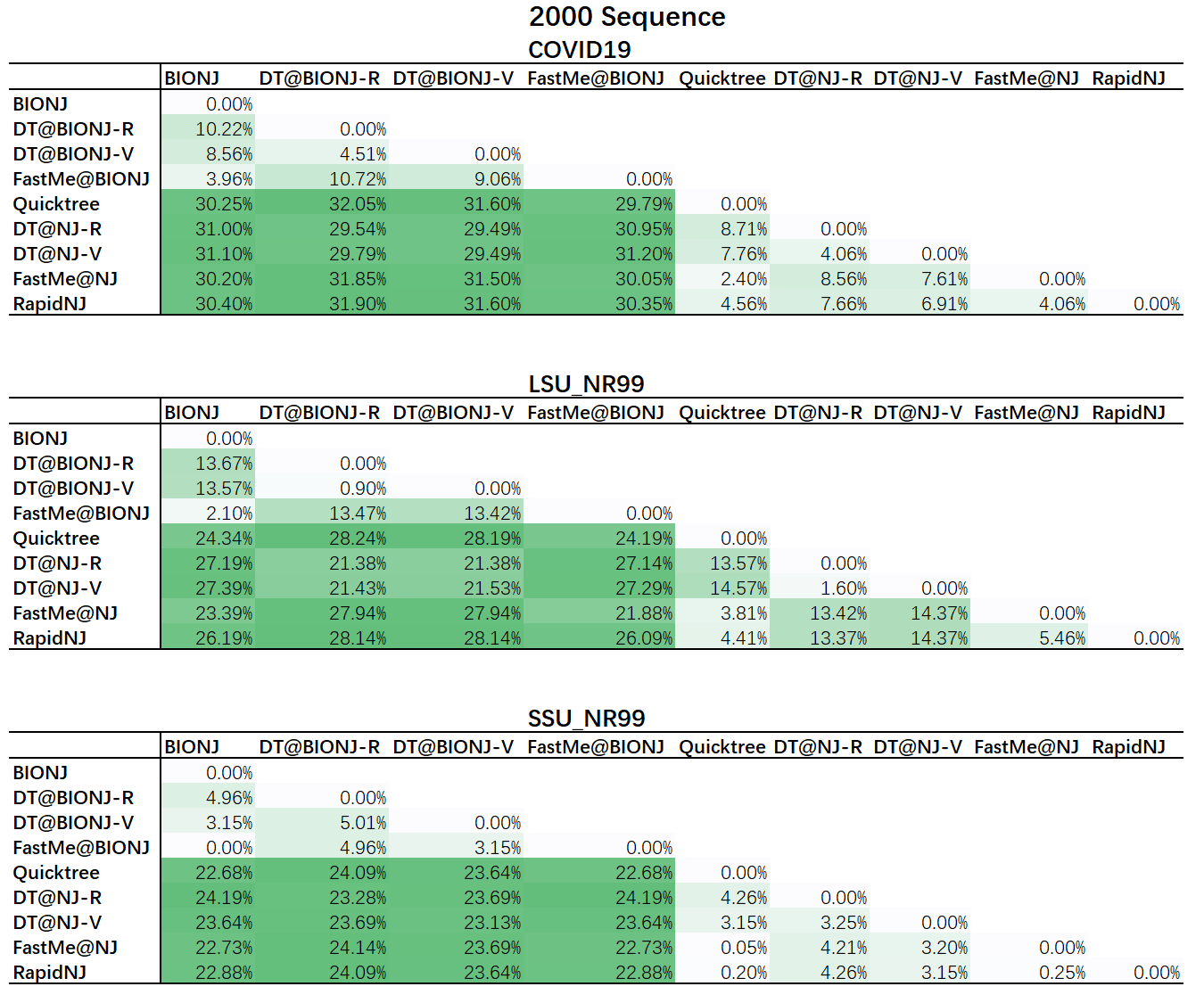


Supplementary Figure S9. The Robinson-Foulds (RF) distances between different trees on the 2,000 sequence datasets. The RF distances were normalized by dividing the current RF by 2(n-3), where n = #taxa. The implementations with higher differences are colored green.


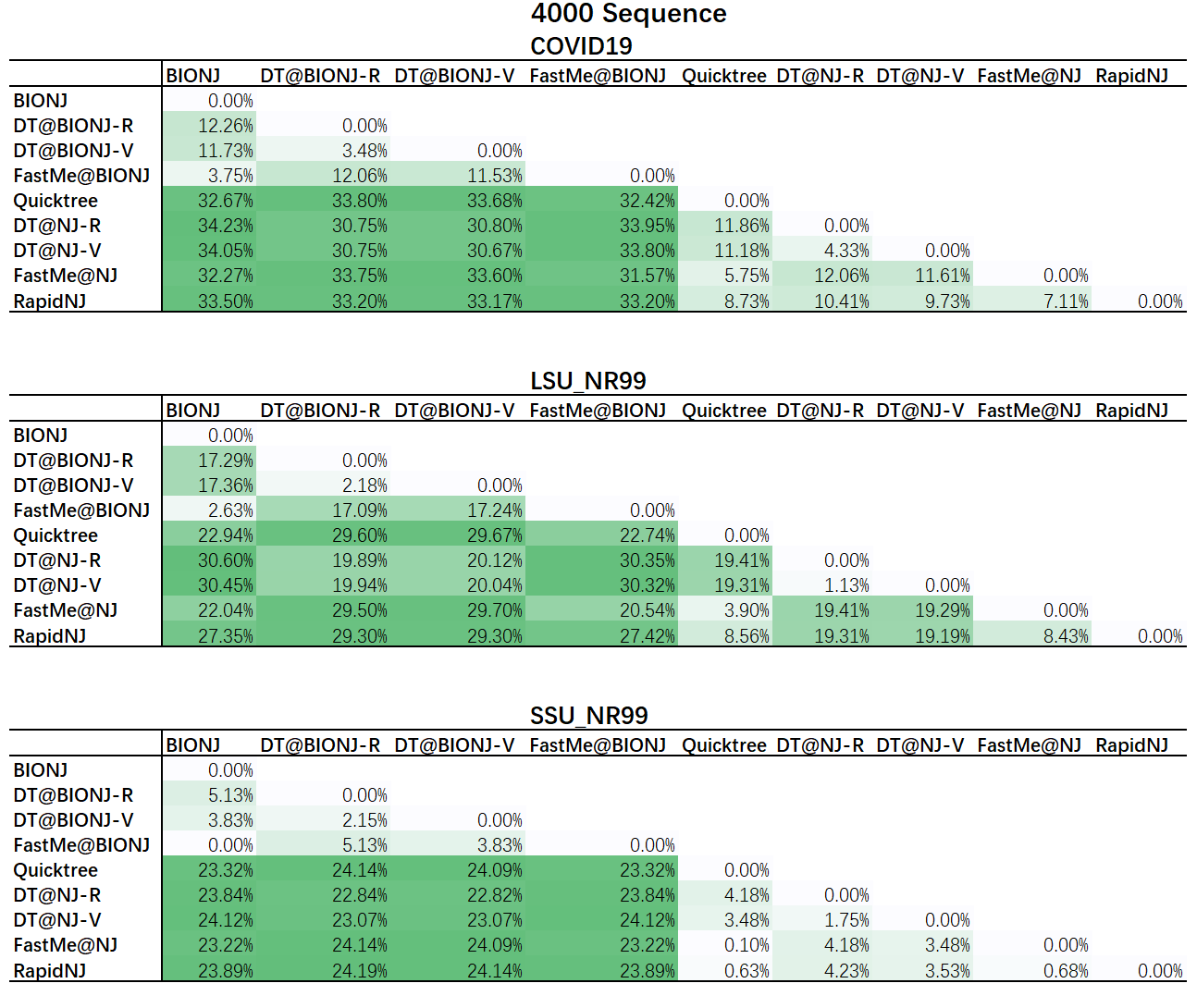


Supplementary Figure S10. The Robinson-Foulds (RF) distances between different trees on the 4,000 sequence datasets. The RF distances were normalized by dividing the current RF by 2(n-3), where n = #taxa. The implementations with higher differences are colored green.


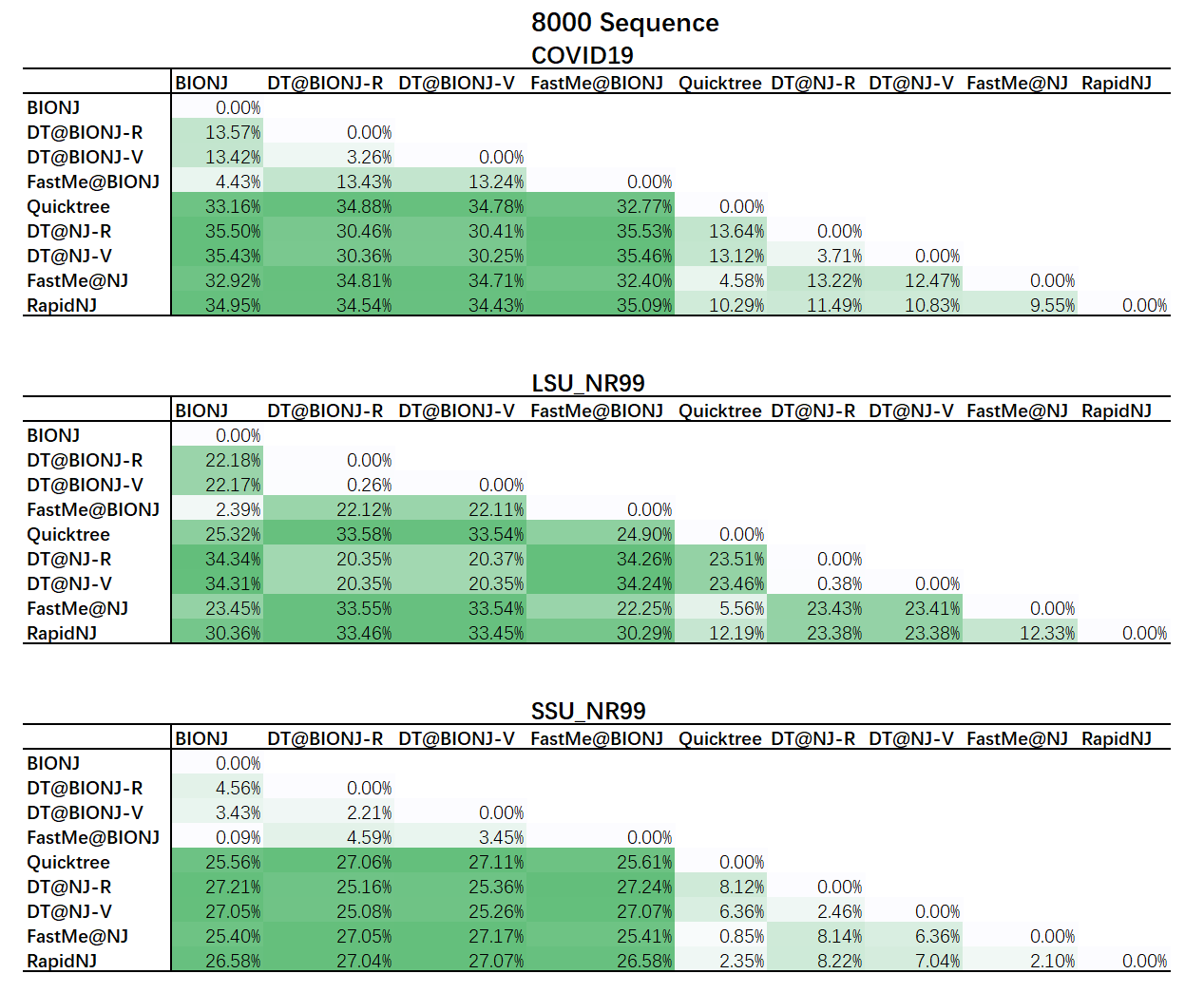


Supplementary Figure S11. The Robinson-Foulds (RF) distances between different trees on the 8,000 sequence datasets. The RF distances were normalized by dividing the current RF by 2(n-3), where n = #taxa. The implementations with higher differences are colored green.


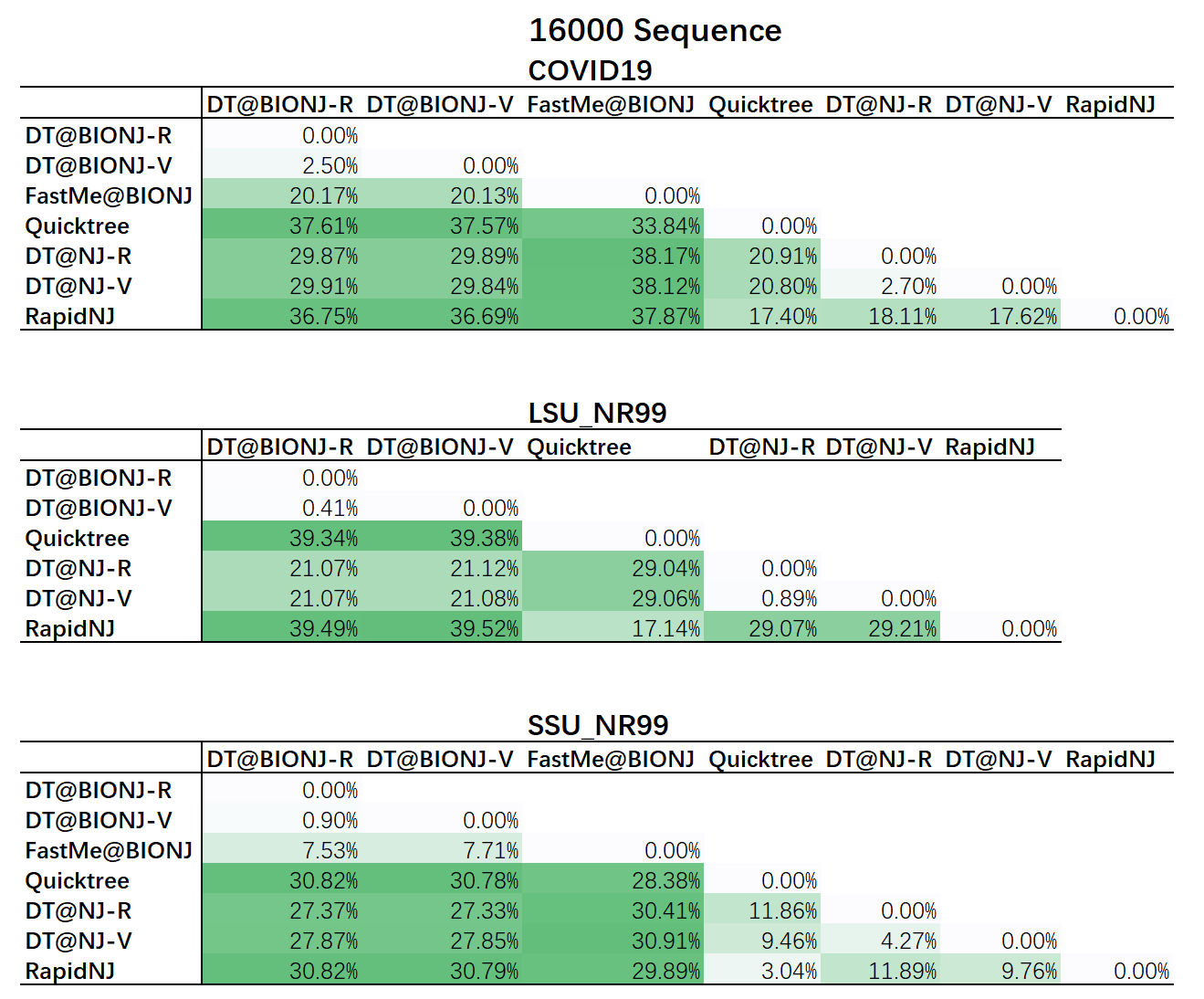


Supplementary Figure S12. The Robinson-Foulds (RF) distances between different trees on the 16,000 sequence datasets. The RF distances were normalized by dividing the current RF by 2(n-3), where n = #taxa. The implementations with higher differences are colored green.


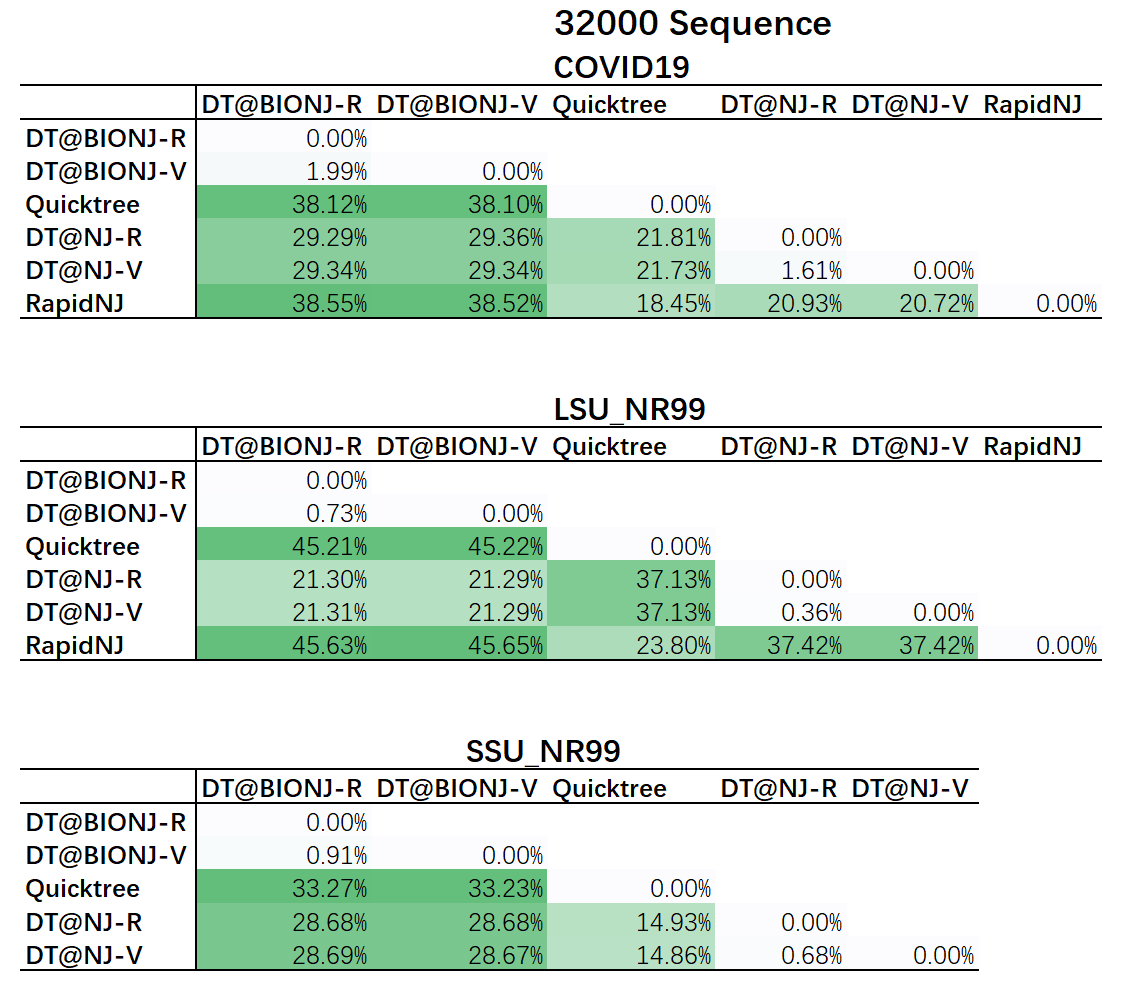


Supplementary Figure S13. The Robinson-Foulds (RF) distances between different trees on the 32,000 sequence datasets. The RF distances were normalized by dividing the current RF by 2(n-3), where n = #taxa. The implementations with higher differences are colored green.


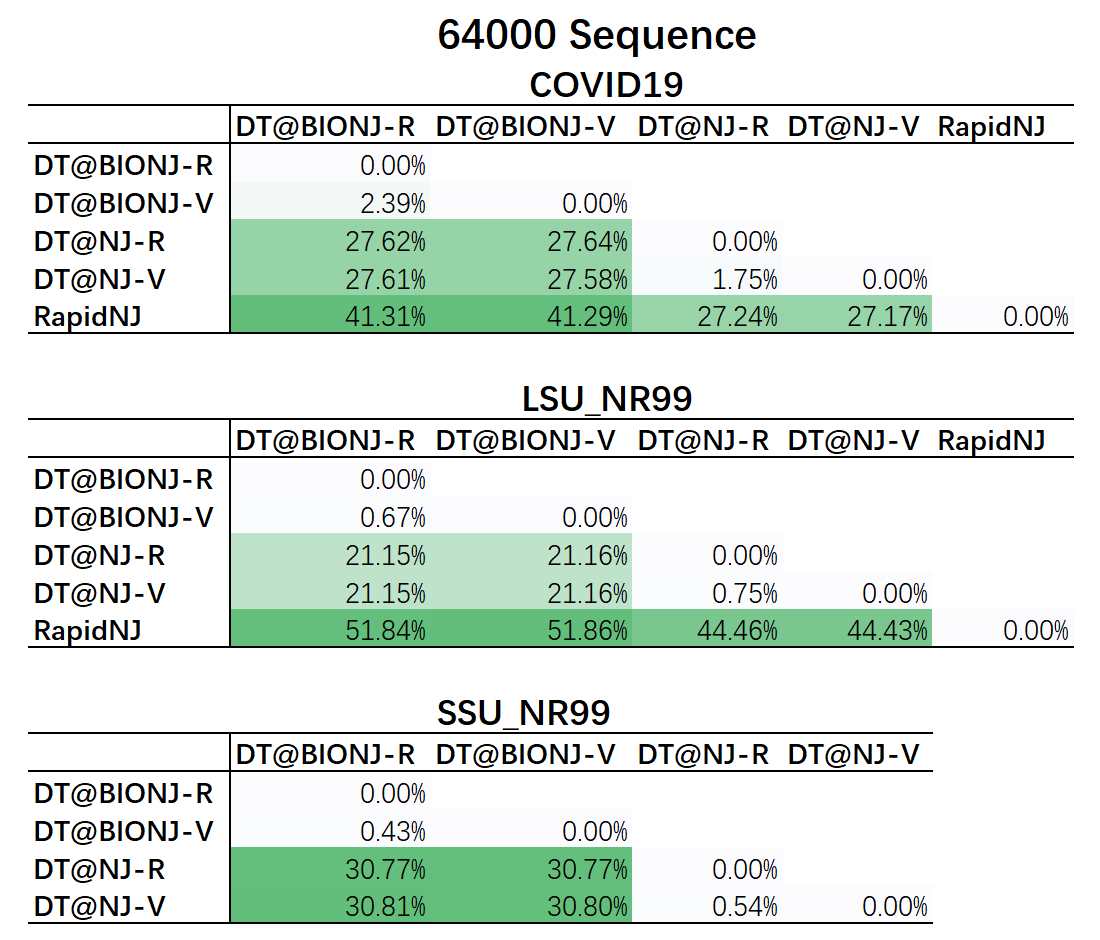


Supplementary Figure S14. The Robinson-Foulds (RF) distances between different trees on the 64,000 sequence datasets. The RF distances were normalized by dividing the current RF by 2(n-3), where n = #taxa. The implementations with higher differences are colored green.


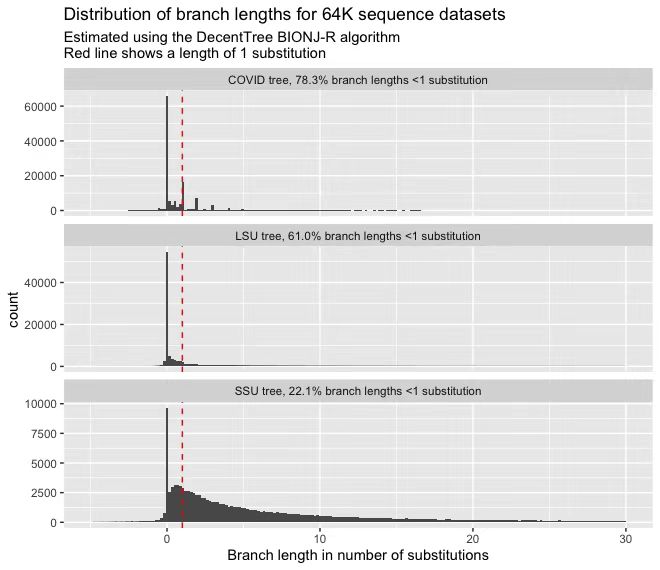


Supplementary Figure S15. The distribution of branch lengths for 64,000 sequence datasets. This figure was created by loading each tree into R and multiplying the branch lengths by the number of sites in each alignment. The figure shows that the most common branch length for each dataset corresponds to zero substitutions (reflecting the large number of identical sequences in the data). The red line shows a branch length of 1 substitution, below which there is very little information with which to confidently resolve branches in a tree. This preponderance of very short branches explains why the Robinson Foulds distances between pairs of trees estimated from the same dataset can be very large for these alignments.

A.


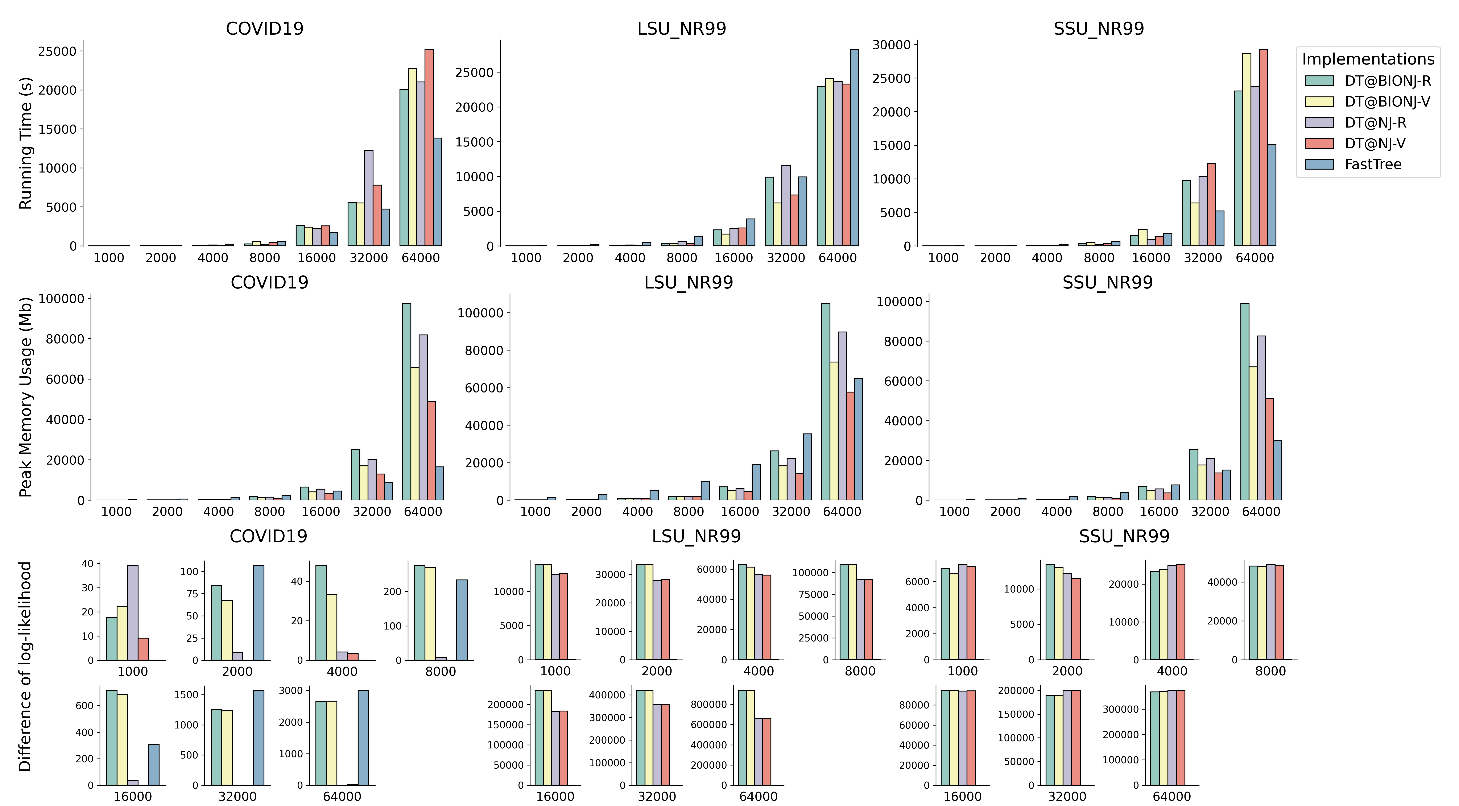


B.


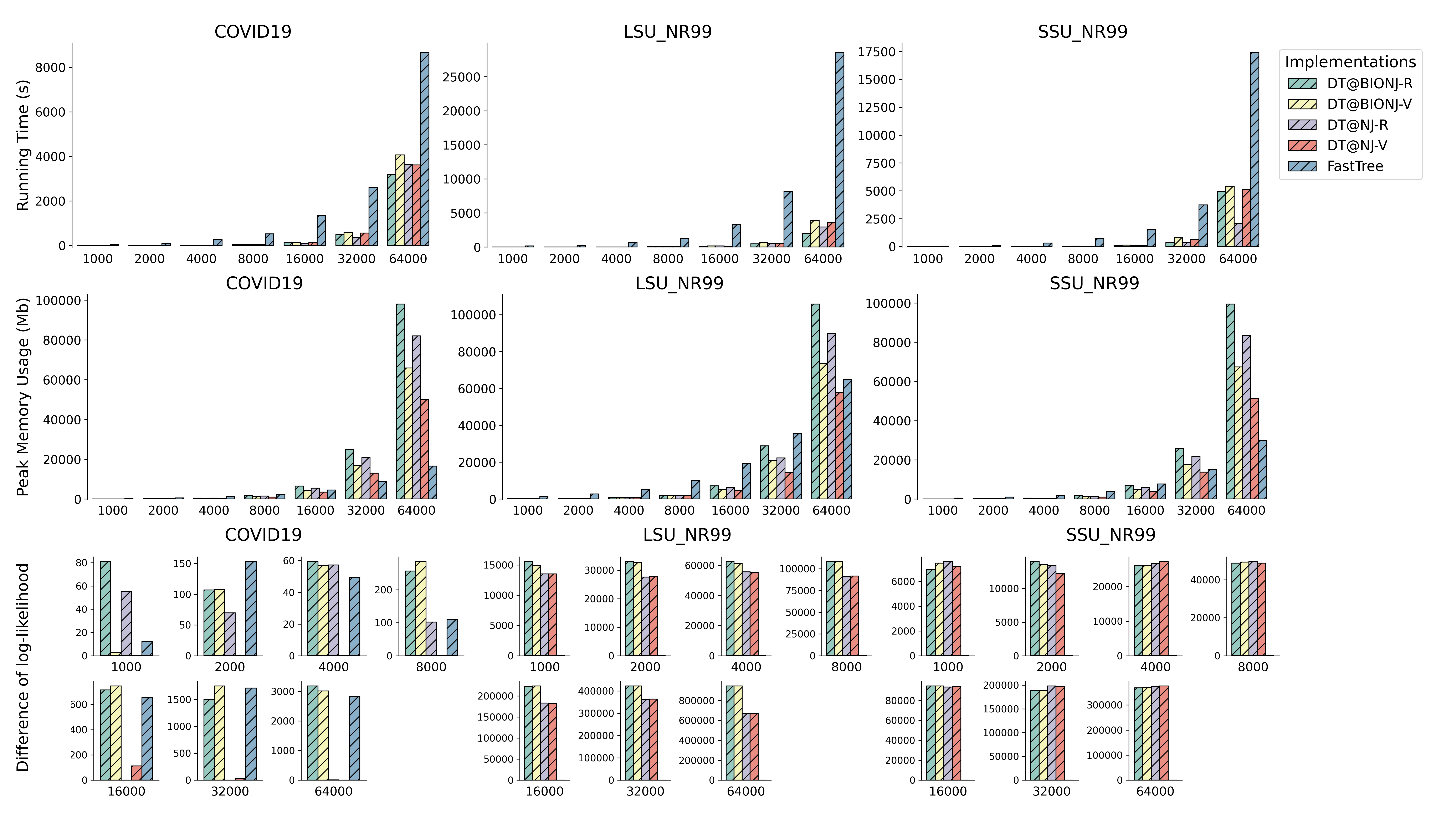


Supplementary Figure S16. Comparing running time, peak memory usage, and difference of log-likelihood between DecentTree and FastTree. The log-likelihood difference is the difference in log-likelihood between each implementation and the implementation with the maximum log-likelihood. A. Using 1 thread. B. Using 32 threads. The 1,000, 2,000, 4,000, 8,000, 16,000, 32,000, and 64,000 sequence subsets of COVID19, LSU_NR99, and SSU_NR99 datasets were compared. With 32 threads, DecentTree was faster than FastTree.


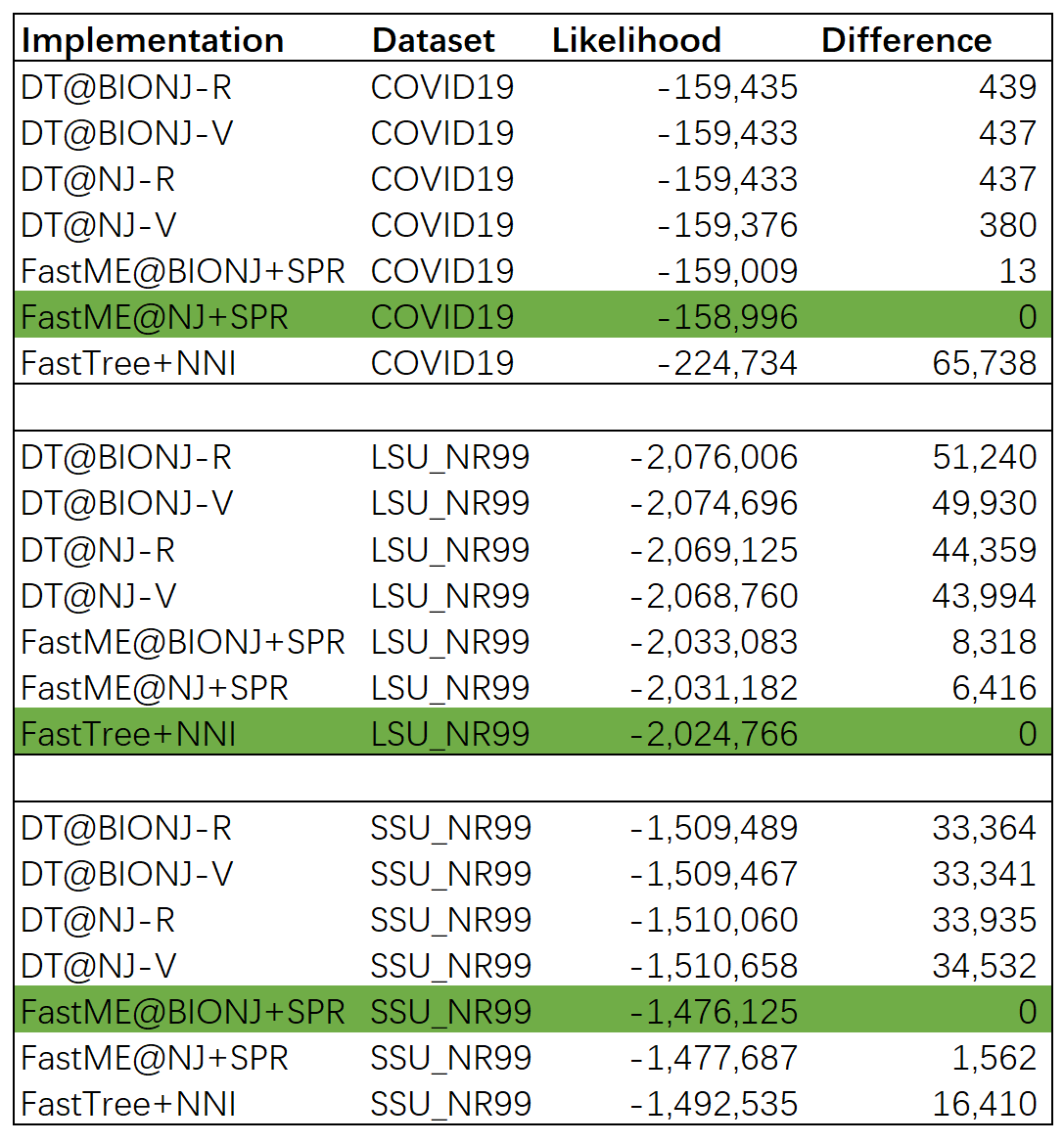


Supplementary Figure S17. The delta plot of inferred tree log-likelihood (GTR+G model) comparison of different implementations on 4,000 sequence subsets of COVID19, LSU_NR99, and SSU_NR99 datasets. FastME was run with subtree pruning and regrafting (SPR), while FastTree was run with nearest neighbor interchange (NNI) to improve the accuracy. DecentTree was run with default settings. The difference is the difference in log-likelihood between each implementation and the implementation with the maximum log-likelihood. The best log-likelihood on each subset was colored green. For the COVID19 and SSU_NR99 subsets, FastME@BIONJ+SPR has the best log-likelihood, whereas FastTree+NNI has the best log-likelihood on the LSU_NR99 subset.

# 3 Supplementary Table

Supplementary Table S1. The running time (hour) of DecentTree and RapidNJ in 64,000 sequence subsets of COVID19, LSU_NR99, and SSU_NR99 datasets. RapidNJ quit without producing any output for SSU_NR99 subset. The fastest DecentTree implementation (DJ@NJ-R) was highlighted.

|  | COVID19 dataset | | LSU dataset | | SSU dataset | |
| --- | --- | --- | --- | --- | --- | --- |
|  | 1 thread | 32 threads | 1 thread | 32 threads | 1 thread | 32 threads |
| DT@NJ-R | **3.47** | **1.12** | **2.48** | **1.08** | **3.10** | **0.94** |
| DT@NJ-V | 4.13 | 1.33 | 2.67 | 1.16 | 5.65 | 1.76 |
| DT@BIONJ-R | 3.72 | 1.26 | 2.59 | 1.11 | 3.38 | 1.05 |
| DT@BIONJ-V | 4.86 | 1.60 | 2.91 | 1.33 | 5.94 | 1.92 |
| RapidNJ | 6.41 | 6.23 | 4.64 | 4.60 | N/A | N/A |
